# Cross‐Cultural Adaptation and Psychometric Assessment of the Persian Version of the FACIT‐Pal Quality of Life Instrument: A Study Protocol

**DOI:** 10.1002/hsr2.72068

**Published:** 2026-04-05

**Authors:** Arash Mojahedi Mohammadi, Arvin Mirshahi, Paul Cella, Behrooz Mahmoodi‐Bakhtiari, Marie Bakitas, Gulcan Bagcivan, Arefe Sayyar, Jason Bredle, Amir Asgari, Maryam Karbasi‐Motlagh

**Affiliations:** ^1^ Students’ Scientific Research Center, Department of Medical‐Surgical Nursing, School of Nursing and Midwifery Tehran University of Medical Sciences Tehran Iran; ^2^ Center for Palliative and Supportive Care University of Alabama at Birmingham Birmingham AL USA; ^3^ FACIT.org, Ponte Vedra Beach FL USA; ^4^ Department of Performing Arts, College of Fine Arts University of Tehran Tehran Iran; ^5^ School of Nursing, and Department of Medicine, Division of Gerontology, Geriatrics, and Palliative Care University of Alabama at Birmingham Birmingham AL USA; ^6^ College of Nursing and Health Sciences, Adult Nursing University of Massachusetts Dartmouth North Dartmouth MA USA; ^7^ Faculty of Social Sciences University of Tehran Tehran Iran; ^8^ Department of Medical Education, Education Development Center, Faculty of Medicine Tehran University of Medical Sciences Tehran Iran

**Keywords:** cross‐cultural adaptation, FACIT‐Pal, Iran, palliative care, Persian, Psychometrics, quality of life, translation

## Abstract

**Background and Aims:**

The growing burden of chronic diseases highlights the need for valid instruments to assess quality of life in palliative care. The Functional Assessment of Chronic Illness Therapy‐Palliative (FACIT‐Pal) measures quality of life in patients with chronic illness receiving palliative care. Although it has been validated in 22 other languages, a Persian version has not yet been translated. This study aims to cross‐culturally adapt and evaluate the psychometric properties of the Persian version of the FACIT‐Pal for Iranian patients.

**Methods:**

This cross‐sectional methodological study is designed to cross‐culturally adapt and evaluate the psychometric properties of the FACIT‐Pal in Iranian individuals with cancer and heart failure. Following FACIT translation guidelines, the process will include two forward translations, reconciliation, backward translation, final review, and qualitative cognitive interviews with 10 patients. Feedback from these patients will be analyzed to further refine the translation. After finalizing the Persian version, 552 Iranian inpatients and outpatients with cancer and heart failure at Imam Khomeini Hospital Complex will be invited to participate after providing written informed consent. Inclusion criteria will be: consent; diagnosis with heart failure or cancer; 18 years of age or older; literacy in Persian. Exclusion criteria will be: diagnosed and documented untreated psychological disorders in their medical record that significantly impair their functioning and ability; having uncorrected communication issues; and completion of less than 80 percent of the meassure. Cronbach's alpha and composite reliability will be used to assess reliability. Construct validity will be examined using confirmatory factor analysis. Statistical tests will be performed using SmartPLS and R software.

**Conclusion:**

The Persian version of the FACIT‐Pal questionnaire is expected to have good psychometric properties, including satisfactory validity and reliability. This instrument could be used as a standard scale to measure the quality of life of patients receiving palliative care in Iran.

**Trial Registration:**

Not applicable.

**Clinical Trial Number:**

Not applicable.

**Abbreviations**
AGFI: Adjusted Goodness of Fit Index
AHA: American Heart Association
CFA: Confirmatory Factor Analysis
CFI: Comparative Fit Index
COSMIN: COnsensus‐based Standards for the Selection of Health Measurement INstruments
EORTC QLQ‐C15‐PAL: European Organization for Research and Treatment of Cancer Quality of Life Questionnaire – Palliative Care
FACIT‐Pal: Functional Assessment of Chronic Illness Therapy – Palliative Care
FACIT‐Pal‐14: Short Form of the Functional Assessment of Chronic Illness Therapy – Palliative Care 14 ‐item version
FACIT‐Sp: Functional Assessment of Chronic Illness Therapy – Spiritual Well‐being
FACT‐G: Functional Assessment of Cancer Therapy – General
FACT‐G7: Functional Assessment of Cancer Therapy – General 7‐item version
GFI: Goodness of Fit Index
IKHC: Imam Khomeini Hospital Complex
MLHF‐Q: Minnesota Living with Heart Failure Questionnaire
PHC: Primary Health Care
RMSEA: Root Mean Square Error of Approximation
SPSS: Statistical Package for the Social Sciences
SRMR: Standardized Root Mean Square Residual
STROBE: Strengthening the Reporting of Observational Studies in Epidemiology
TLI: Tucker–Lewis Index
TUMS: Tehran University of Medical Sciences
WHO: World Health Organization

## 1 Background

Chronic diseases are conditions that may last for at least for 3 months and may worsen over time [1]. The prevalence of chronic diseases has been increasing, making them the major cause of morbidity and resulting in 75% of global mortality in the world each year [2, 3]. Also, a significant portion of annual health expenditure in the USA is attributed to the chronic diseases [4]. In Iran, the aged population is increasing in parallel with the chronic diseases prevalence, such as ischemic heart diseases, stroke, and diabetes, resulting in an estimated 82 percent of mortality [5, 6]. Also, chronic diseases impose a huge burden on the Iranian healthcare workforce [7].

Among chronic conditions, the incidence of cancer is rapidly increasing. According to the American Cancer Society, about 20 million people were diagnosed with cancer in 2022, and this number is anticipated to increase to 35 million by 2050 [8, 9]. Cancer may influence patients’ lives in various aspects and interfere with their quality of life, which makes it a large concern. In a meta‐analysis, it is estimated that quality of life is significantly affected from 2 to 26 years after cancer diagnosis [10]. Therefore, measuring the quality of life among patients suffering from cancer is a key factor in controlling, managing, and treating the disease [11].

In addition to cancer, heart failure is a quite prevalent chronic condition in Iran, and results in debilitating conditions among patients [12]. It is shown that patients with heart failure experience significantly reduced quality of life in Iran [13]. This condition may originate from various complications (like liver and kidney diseases, arrhythmias, or readmissions) or comorbidities, which could severely decrease their quality of life [14, 15].

According to the World Health Organization (WHO), “quality of life is a concept which aims to capture the well‐being, whether of a population or individual, regarding both positive and negative elements within the entirety of their existence at a specific point in time” [16]. Due to the traumatic influence of chronic diseases, measuring quality of life is considered critical in patients with cancer and heart failure [17].

Palliative care, as an approach aimed to alleviate pain and suffering among patients with chronic diseases, can be applied to patients and their families. Ultimately, it seeks to optimize the quality of life for patients and their families [18]. While much research has focused on palliative care for patients with cancer, patients with heart failure also require high‐quality palliative care due to the significant burden of their disease [19]. In the Primary Health Care (PHC) system in Iran, palliative care is not highlighted adequately and is counted as a novel approach to terminally and/or chronically ill patients [20, 21]. The WHO introduces palliative care as an effective strategy to enhance quality of life among individuals with cancer and their families, as it can ease about 90 percent of physical, psychological, and spiritual distress [9]. On the other hand, the American Heart Association (AHA) has pinpointed applying palliative care besides usual care [22]. Bagheri et al. suggest future research focus on core elements of palliative care, such as quality of life [23].

As mentioned above, a primary goal in palliative care is to optimize patients’ quality of life. Thus, in order to measure the effectiveness of palliative interventions, the presence of an accurate, valid, and reliable scale is crucial. Over the past decades, the assessment of quality of life has gained significance to ensure that clinical and care‐related decisions align with patients’ perspectives [24]. In a systematic review, the absence of a comprehensive quality‐of‐life measurement for Iranian patients with cancer has already been reported [25]. One of the most recent questionnaires validated in Iran is the “Minnesota Living with Heart Failure Questionnaire (MLHF‐Q)”. This questionnaire is specifically produced to evaluate the quality of life among the individuals suffering from heart failure. This questionnaire measures patients’ physical, mental, and social conditions using 21 items [26]. However, it is specific to individuals with heart failure and generalizing it to other chronic conditions needs more investigations. Moreover, the FACIT‐Pal (in comparison with the MLHF‐Q) evaluates the quality of life of general chronic illness in palliative care patients. It focuses on Physical well‐being, Social well‐being, Emotional well‐being, and Functional well‐being, and palliative‐specific items [27].

King et al. compared four Quality of life scales: EORTC QLQ‐C15‐PAL, FACT‐G7, FACIT‐Pal and short‐form FACIT‐Pal‐14 in palliative care settings. Among these, FACIT‐Pal had a better internal consistency. Also, FACIT scales had better test–retest reliability [28]. The FACIT scale is considered a reliable and valid measurement worldwide [29]. This scale was developed by Cella and colleagues [30], and managed by the internationally known FACIT organization [31], creating more than 100 scales, some of which have been translated and linguistically validated into over 80 languages [32].

The 27‐item FACT‐G core module, published in 1993, was initially developed for patients with cancer before broadening to include other chronic illnesses [29, 30]. One such broadened instrument included the 19‐item palliative care specific module, which was added to the FACT‐G, resulting in the 46‐item FACIT‐Pal. Items from the questionnaire were based on interviews of patients and their families [27]. Greisinger et al., [33] have interviewed 194 patients suffering from life‐limiting illnesses and measured their concerns in terms of different aspects of quality of life. The palliative care subscale contains items related to signs and symptoms patients experience in the advanced phase of their illness [27, 33]. The FACIT Pal was subsequently tested by Lyons and colleagues (2009) for reliability and validity [27, 29]. One of the advantages of the FACIT‐Pal scale is its dedication to palliative care, ease of administration, and focus on social well‐being in comparison to other, similar measurements. Also, the scale has shown satisfactory reliability and validity in 22 different languages [34, 35]. It has been translated and psychometrically assessed in regions such as Turkey [36], Spain [37], Germany [38], South Africa, Kenya, and Uganda [29], and recently in China [39].

Cultural adaptation is a crucial part of developing correct and effective principles for implementing care programs in different communities [40]. Despite having satisfactory reliability and validity in multiple languages, the FACIT‐Pal scale has not yet been translated into Persian and assessed in Iranian culture. Given the special linguistic and cultural characteristics of the Persian‐speaking population, strict cultural adaptation and psychometric testing of the FACIT‐Pal scale are absolutely necessary for its accurate implementation in the Iranian palliative care context. Thus, this study primarily aims to cross‐culturally adapt the FACIT‐Pal scale into Persian using a forward‐backward translation method and cognitive interviews (in accordance with the FACIT guidelines), and ensure its equivalence with its original English version, and assess its psychometric properties.

## 2 Methods

### 2.1 Study Design and Setting

**Figure 1:**
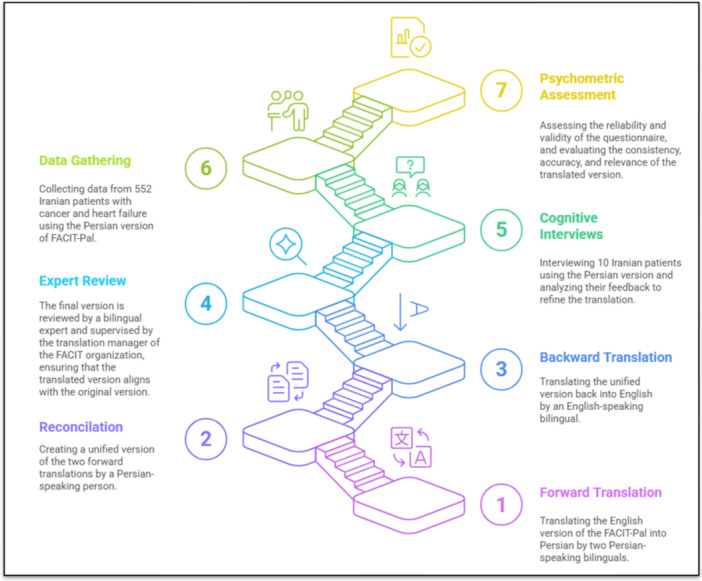
The brief illustration of the study process.

This methodological study will be done cross‐sectionally at the Imam Khomeini Hospital Complex (IKHC) affiliated with the Tehran University of Medical Sciences (TUMS). IKHC is the largest tertiary referral hospital in Iran, comprising Vali‐e‐Asr Hospital, Imam Khomeini Hospital, and the Cancer Institute. Together, they have nearly 400 faculty members, about 4000 administrative and clinical staff, and more than 1000 active beds [41]. Inpatients or outpatients diagnosed with heart failure or cancer, who meet the inclusion and exclusion criteria, will be invited to participate. The protocol of this study is approved by the Research Ethics Committees of TUMS (IR. TUMS. MEDICINE. REC.1403.432) and patients will be asked to provide written informed consent. This study aims to assess the psychometric properties of the Persian version of the FACIT‐Pal scale and will be carried out in two main phases. This protocol adheres to the STROBE (Strengthening the Reporting of Observational studies in Epidemiology) and COSMIN guidelines [42, 43]. The study process is briefly illustrated in Figure 1.

### 2.2 Phase 1: Cross‐Cultural Adaptation and Harmonization

The copyright of the FACIT questionnaires entirely belongs to the FACIT organization. This study will be conducted after obtaining the written permission letter from the FACIT organization, using the standardized multilingual and validation measurement system [44, 45]. This ensures that the translation of the FACIT‐Pal questionnaire into Persian contains the sufficient quantitative criteria equivalent to the original version of FACIT‐Pal [46]. The written permission was obtained from FACIT.org on September 2, 2024.

The translation phase consists of two forward translations, one reconciliation of the two forward translations, and one backward translation. Initially, two bilingual Iranians—literate in English—will translate the English version to Persian (Forward translation). Afterward, another Persian‐speaking person makes a unified version of the Persian version out of the two forward translations. This unified version is then translated into English by an English‐speaking bilingual who is literate in Persian. If no translation issues are identified, an expert in the field of medical sciences will review the final version of the Persian translation and, if there are any content problems, will express his/her opinions to the team leader.

Once the Persian translation is finalized, cognitive structured interviews will be conducted based on the organization's standard guidelines. This will involve one experienced researcher who is familiar with the questionnaire, palliative care, and qualitative studies, and who has cognitive interview experience. Ten Persian‐speaking patients will complete the FACIT measure and their feedback will be analyzed to further refine the translation, if necessary. After the final application of the patients’ comments, the process of translation and harmonization of the questionnaire will be completed. It is noteworthy that all these steps will be supervised by Paul Cella, the Translations Manager of the FACIT organization, and the principal investigator of the research team, Arvin Mirshahi, will remain in contact with him throughout the translation process.

### 2.3 Phase 2: Data Gathering & Psychometric Assessment

#### 2.3.1 Data Gathering

##### 2.3.1.1 Participants

The study sample will be divided into two groups: (1) Patients with heart failure, comprising 50 percent (276 patients) of the total sample size, will be recruited from CCU, cardiology wards, and the heart failure clinic of IKHC; and (2) Patients with cancer: comprising 50 percent (276 patients) of the total sample size, will be recruited from the cancer institute (oncology ward) of IKHC and the cancer clinics of IKHC. The inclusion criteria will be: (1) consent to participate in the study; (2) diagnosed with heart failure or cancer; (3) 18 years of age or older; (4) having the literacy to read and write in Persian. The exclusion criteria will be: (1) presence of a documented, untreated severe psychological disorder recorded in the medical record by a psychiatrist or treating physician, including schizophrenia spectrum disorders, bipolar disorder, or active substance use disorder, which is considered to cause significant functional impairment, defined as impaired judgment, cognition, or communication that interferes with the individual's capacity to understand study procedures or complete study assessments; (2) uncorrected communication impairments (auditory, visual, or verbal) that preclude participation; and (3) completion of less than 80% of the study measures.

### 2.4 Questionnaire Design

The questionnaire, which is given to the patients, consists of two core parts:

A. Sociodemographic and Clinical Characteristic questionnaire: This part of the questionnaire will aim to know the target population in a more detailed way. This section consists of two parts: (1) sociodemographic characteristics, such as age, gender, level of education, marital status, occupation, and income; (2) clinical characteristics, such as type of disease, heart failure classification (in case of heart failure), type of cancer, metastasis (in case of cancer), type of referral (inpatient or outpatient), length of hospitalization, and comorbidities.
B. FACIT‐Pal Quality of Life Questionnaire: The psychometric properties of this instrument were assessed in 2009 by Lyons et al. [27] via a randomized clinical trial study. The FACIT instrument is known as a comprehensive instrument for measuring the quality of life of patients with chronic diseases, which includes 27 items from the FACT‐G questionnaire and the palliative care subscale (FACIT‐Pal).

In the study by Lyons et al. [27], the psychometric properties of the 46‐item FACIT‐Pal were examined in English. The 19 items—which are specifically about palliative care—were developed based on interviews conducted with patients with advanced cancer. After adding the palliative care section and examining the internal reliability with Cronbach's alpha coefficient, the palliative care section of this questionnaire obtained a Cronbach's alpha of 0.82 and in general, the Cronbach's alpha of the other domains of the English questionnaire obtained a Cronbach's alpha between 0.75 and 0.93. This questionnaire includes a total of 46 items, which are scored on a 5‐point Likert scale. The options for these questions are scored on a 5‐point Likert scale ranging from 0 to 4, and the respondent will receive a total score between 0 and 184. Higher scores indicate a higher quality of life score [27, 36]. In the present study, the FACIT‐Pal questionnaire will be translated into Persian based on the implementation of FACIT's methodology.

### 2.5 Sampling Process

Among the individuals with cancer and heart failure referred to the inpatient wards and heart failure and cancer outpatient clinics of the IKHC, 552 patients will be selected as the research sample by the convenience sampling method, based on inclusion and exclusion criteria. Of the desired sample size, 50% will be allocated to patients with heart failure (276 patients) and 50% to patients with cancer (276 patients). The necessary matters regarding the study process, the objectives of the research, as well as the confidentiality of their information, will be explained to the patients, and they will be invited to participate in the present study if they wish. Written informed consent will be obtained, and the research instruments, including the demographic questionnaire and the FACIT‐Pal questionnaire, translated into Persian, will be provided. Patients will be asked to complete the questionnaire using a self‐report approach.

### 2.6 Sample Size

It is suggested to include 5 to 10 subjects per item for psychometric assessment studies [47]. According to this recommendation, it was decided to include 10 patients for each item on the 46‐item FACIT‐Pal questionnaire, resulting in a total requirement of 460 patients to achieve the maximum sample size. After considering a 20 percent attrition rate, the final sample size is determined to be 552 patients (276 diagnosed with heart failure and 276 diagnosed with cancer).

#### 2.6.1 Psychometric Assessment

Following the translation process, internal consistency reliability of the instrument will be examined by the computation of Cronbach's alpha and McDonald's omega coefficients. These coefficients will be computed in SPSS software and will be interpreted as weak for estimates of ~ 0.5, needs revision for estimates between 0.5 and 0.7, and adequate for estimates at 0.7 or higher.

To examine the evidence of the structural validity of the instrument, covariance‐based confirmatory factor analysis (CFA) will be implemented using software such as the lavaan package in R or covariance‐based modeling routines in SmartPLS. To assess the goodness of fit of the measurement model, a number of commonly used fit indices [48] will be used including RSMEA (good fit < 0.06), SRMR (good fit ~ < 0.08), CFI, GFI, AGFI, and TLI (good fit ~ > 0.9). Known‐groups validity will be examined by comparing FACIT‐Pal scores by disease stage within patient groups (i.e., Heart failure classification and metastatic status will be obtained from medical records and verified with clinician approval) using ANOVA.

Reliability coefficients will be computed overall and examined separately for cancer and heart failure subgroups. Measurement invariance [48] between cancer and heart failure patients will be examined with multiple‐group analyses, sequentially fitting and comparing CFA model fit for (1) configural invariance (i.e., the same factor structure is imposed on both patient groups), (2) weak invariance (i.e., the factor loadings are constrained to be equal across the patient groups), and (3) strong invariance (i.e., the factor loadings and intercepts are constrained to be equal across the patient groups).

## 3 Discussion

This study provides a protocol for cross‐cultural adaptation and psychometric assessment of the FACIT‐Pal scale in the Persian‐speaking population. The FACIT‐Pal scale is being used to measure the quality‐of‐life and palliative care intervention outcomes in several languages, such as Spanish [37], German [38], Turkish [36], African languages [29], and recently in Chinese [39].

Bagcivan et al. have translated FACIT‐Pal into Turkish and assessed its psychometric properties [36]. Their research has shown that the FACIT‐Pal‐TR owns strong psychometric validity and reliability. The Cronbach's alpha coefficient in the Turkish version was 0.932 and also test–retest scores had shown a significant relationship (*r* = 0.877, *p* < 0.001). As the Iranian and Turkish people have a lot in common in terms of religion, history, and culture [49], it is anticipated that the Persian version of FACIT‐Pal will have the same strength in terms of psychometric properties.

Additional research demonstrating the potential for the FACIT‐Pal questionnaire has been conducted by Mirshahi et al.'s team who translated and psychometrically validated the FACIT‐Pal‐14—the shortened version of 46‐item FACIT‐Pal—in Persian to measure the patient's quality‐of‐life within a randomized controlled trial. Despite the focus of most articles on measuring the quality‐of‐life in cancer patients, in this article, patients with heart failure have also been included [50]. This is a promising potential for the Persian version of the 46‐item FACIT‐Pal to be an applicable scale for measuring QoL. In Iran, palliative care is mainly focused on cancer and specifically on the physical aspect of the patients; whereas this research shows the feasibility of the FACIT‐Pal‐14 validation among Iranian people suffering from heart failure [51].

In the Iranian context, there are alternative FACIT scales with different subscales. Out of these, the Persian version of FACIT‐Sp has been validated in 2013 by Jafari et al. the goal of their study was to translate and investigate the reliability and validity of the Persian version of the FACIT‐Sp among Muslim Iranians in treatment for cancer. The Cronbach's alpha coefficient in the Persian version of FACIT‐Sp ranged from 0.72 to 0.90, which showed sufficient reliability [52]. Similar to this study, our research will be conducted among Iranian patients who are predominantly Muslim. However, the focus of this study was limited to patients diagnosed with cancer.

The concept of palliative care is not sufficiently understood by the public and also health‐care staff, which has led to a lack of knowledge about its beneficial outcomes [50]. A reliable and valid tool for measuring quality of life is essential for evaluating the quality of services, the effectiveness of care and treatments, and the impact of interventions [53]. Also, it is mentioned that the community and cultural context could significantly impact the feasibility of the interventions that are implemented, and this shows how culture could be tremendously effective [40]. Moreover, the lack of a gold standard questionnaire, along with a variety of quality‐of‐life scales, has made it difficult to make a comprehensive evaluation of quality‐of‐life [53]. The FACIT‐Pal instrument has yet to be translated into Persian and assessed psychometrically in Iranian culture. Thus, this study could be an outstanding step approaching the optimum quality‐of‐life in Iranian people receiving palliative care.

### 3.1 Limitations

Using a self‐report data collection method may introduce potential response bias. To minimize this risk, the researcher will provide clarification for any ambiguous questionnaire items to ensure that participants fully understand the content. Nevertheless, the possibility of social desirability bias and recall bias cannot be completely ruled out. Another limitation concerns the generalizability of the findings. This study uses a single‐center sample recruited exclusively from the Imam Khomeini Hospital Complex, a major tertiary referral center. Although patients with cancer and heart failure—two of the most common chronic disease groups—were selected to enhance generalizability, the results may not be representative of patients in rural, non‐academic, or community‐based settings. Future multi‐center validation studies are recommended. Furthermore, the cross‐sectional design of this study does not allow for assessment of test–retest reliability, and therefore temporal stability of the Persian FACIT‐Pal remains unknown. In addition, although the cognitive interview sample of 10 participants follows FACIT translation and linguistic validation guidelines, it may not fully capture Iran's linguistic, cultural, and dialectical diversity. This limited sample size may affect the comprehensiveness of cognitive debriefing, and future studies with larger and more diverse samples are warranted.

## Author Contributions

Arvin Mirshahi envisioned the idea and shaped the methodology of the study with support from Marie Bakitas, Jason Bredle, Gulcan Bagcivan, Behrooz Mahmoodi‐Bakhtiari, and Maryam Karbasi‐Motlagh. Arvin Mirshahi took the lead in gathering team members to write the research proposal and this protocol manuscript. Arash Mojahedi Mohammadi wrote the research proposal with assistance from Arefe Sayyar and Arvin Mirshahi. Jason Bredle is the Director, and Paul Cella is the Translations Manager at FACIT. org, who will work closely with the research team to translate the tool according to the high‐quality standards of the FACIT. org translation method. Amir Asgari has been involved in revising the final manuscript and integration of research methodology into the paper structure. All authors have read and approved the final version of the manuscript. Arvin Mirshahi and Maryam Karbasi Motlagh have full access to all the data in this study and take complete responsibility for the integrity and accuracy of the data analysis.

## Funding

The authors have nothing to report.

## Ethics Statement

The study protocol was approved by the Institutional Review Board at Tehran University of Medical Sciences (Code: IR. TUMS. MEDICINE. REC.1403.432). All participants will provide written informed consent.

## Consent for Publication

The authors have nothing to report.

## Conflicts of Interest

The authors declare no conflicts of interest.

## Transparency Statement

The lead authors, Arvin Mirshahi and Maryam Karbasi‐Motlagh, affirm that this manuscript is an honest, accurate, and transparent account of the study being reported; that no important aspects of the study have been omitted; and that any discrepancies from the study as planned (and, if relevant, registered) have been explained.

## Data Availability

No datasets were generated or analyzed during the current study.
